# Alternative Splicing of Three Genes Encoding Mechanotransduction-Complex Proteins in Auditory Hair Cells

**DOI:** 10.1523/ENEURO.0381-20.2020

**Published:** 2021-02-23

**Authors:** Zijing Zhou, Xiaojie Yu, Biaobin Jiang, Wanying Feng, Ye Tian, Zhiyong Liu, Jiguang Wang, Pingbo Huang

**Affiliations:** 1 Division of Life Science, Hong Kong University of Science and Technology, Hong Kong, China; 2 Department of Chemical and Biological Engineering, Hong Kong University of Science and Technology, Hong Kong, China; 3 State Key Laboratory of Molecular Neuroscience, Hong Kong University of Science and Technology, Hong Kong, China; 4 HKUST Shenzhen Research Institute, Hong Kong University of Science and Technology, Hong Kong, China; 5 Hong Kong Branch of Guangdong Southern Marine Science and Engineering Laboratory (Guangzhou), Hong Kong University of Science and Technology, Hong Kong, China; 6 Bioscience and Biomedical Engineering Thrust, The Hong Kong University of Science and Technology (Guangzhou), Hong Kong, China; 7 Institute of Neuroscience, Chinese Academy of Science, Shanghai, China

**Keywords:** alternative splicing, auditory hair cell, ion channel, mechanosensation, tonotopic gradient

## Abstract

The mechanotransduction (MT) complex in auditory hair cells converts the mechanical stimulation of sound waves into neural signals. Recently, the MT complex has been suggested to contain at least four distinct integral membrane proteins: protocadherin 15 (PCDH15), transmembrane channel-like protein 1 (TMC1), lipoma HMGIC fusion partner-like 5 (LHFPL5), and transmembrane inner ear protein (TMIE). However, the composition, function, and regulation of the MT-complex proteins remain incompletely investigated. Here, we report previously undescribed splicing isoforms of TMC1, LHFPL5, and TMIE. We identified four alternative splicing events for the genes encoding these three proteins by analyzing RNA-seq libraries of auditory hair cells from adult mice [over postnatal day (P)28], and we then verified the alternative splicing events by using RT-PCR and Sanger sequencing. Moreover, we examined the tissue-specific distribution, developmental expression patterns, and tonotopic gradient of the splicing isoforms by performing semiquantitative and quantitative real-time PCR (qRT-PCR), and we found that the alternative splicing of TMC1 and LHFPL5 is cochlear-specific and occurs in both neonatal and adult mouse cochleae. Our findings not only reveal the potential complexity of the MT-complex composition, but also provide critical insights for guiding future research on the function, regulation, and trafficking of TMC1, LHFPL5, and TMIE and on the clinical diagnosis of hearing loss related to aberrant splicing of these three key genes in hearing.

## Visual Abstract

**Figure:**
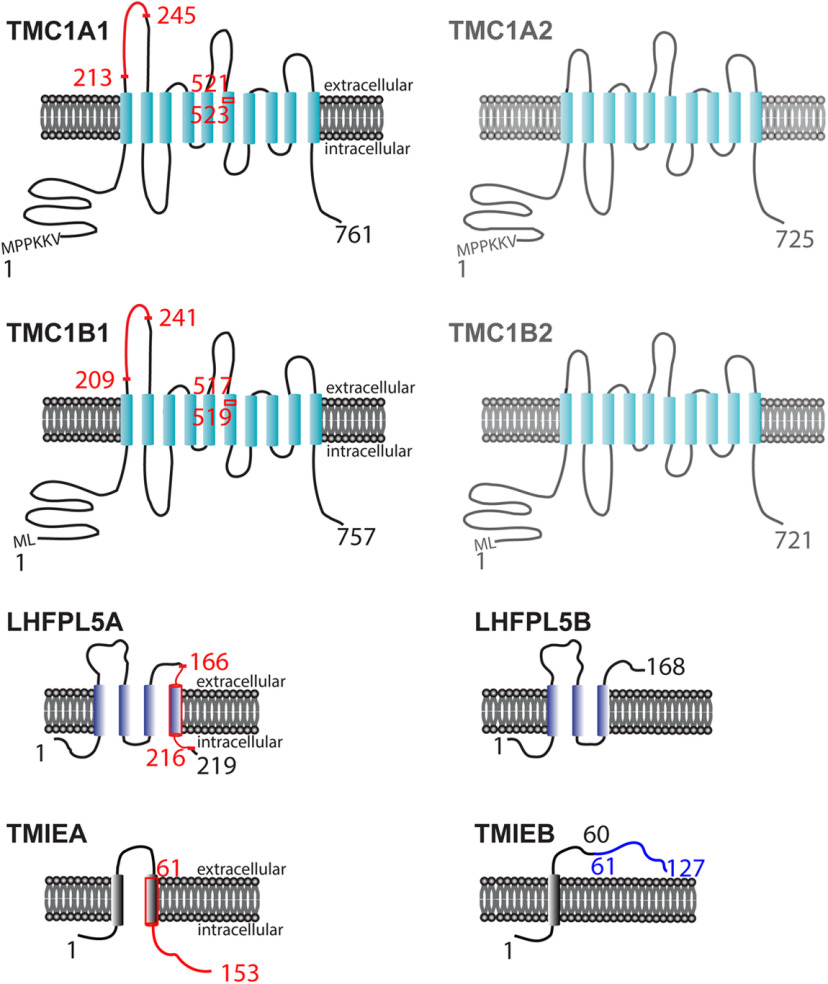

## Significance Statement

We have identified previously unreported splicing variants of transmembrane channel-like protein 1 (TMC1), lipoma HMGIC fusion partner-like 5 (LHFPL5), and transmembrane inner ear protein (TMIE), the pivotal molecules forming the hair-cell mechanotransduction (MT) machinery in the inner ear. Our findings reveal the potential complexity of the MT-complex composition and provide valuable guidance for future research on the function, regulation, and trafficking of TMC1, LHFPL5, and TMIE. Furthermore, our study could help direct the clinical diagnosis of hearing loss related to aberrant splicing of TMC1, LHFPL5, and TMIE.

## Introduction

The mechanotransduction (MT) complex is a channel-containing macromolecular complex in auditory hair cells that converts the mechanical stimulation of sound waves into neural signals in auditory perception. The precise molecular composition of this macromolecular transducer remains incompletely established, but the MT complex has been suggested to contain at least four membrane proteins: transmembrane channel-like protein 1 (TMC1), protocadherin 15 (PCDH15), lipoma HMGIC fusion partner-like 5 (LHFPL5), and transmembrane inner ear protein (TMIE). The human orthologs of TMC1 and TMIE were first identified as products of genes associated with nonsyndromic hearing loss (Kurima et al., 2002; Naz et al., 2002), and LHFPL5 was first reported as the protein whose mutation caused deafness in hurry-scurry mice (Longo-Guess et al., 2005). TMC1 and TMIE were recently identified as components of the MT channel (Kawashima et al., 2011; Kim and Fettiplace, 2013; Pan et al., 2013, 2018; Cunningham et al., 2020), PCDH15 forms part of the tip link that is generally accepted to serve as the gating spring of the MT channel (Ahmed et al., 2006; Bartsch et al., 2019), and LHFPL5 is considered to functionally couple the tip link to the MT channel and stabilize TMC1 expression (Xiong et al., 2012; Beurg et al., 2015; Yu et al., 2020). Moreover, these MT-channel components have been found to display specific spatiotemporal patterns of expression. For example, TMC1 mRNA is specifically expressed in the cochlea (and several other tissues; Keresztes et al., 2003) and also exhibits a specific temporal pattern, it is first detected at postnatal day (P)4 and is maintained through adulthood in the cochlea (Kawashima et al., 2011); moreover, TMC1 protein expression gradually increases along the tonotopic axis from the apex to the base in outer hair cells (OHCs; Beurg et al., 2018). Despite these recent notable advances in our understanding of the MT complex, the composition, function, and regulation of the MT-complex proteins remain to be comprehensively investigated.

Alternative splicing, which occurs in ∼95% of multiexonic genes in humans (Pan et al., 2008), substantially increases the diversity of the proteins that can be encoded by the genome, and this protein diversity contributes to tissue-identity acquisition, organ development, and tissue and organ physiology (Baralle and Giudice, 2017; Liu et al., 2017). For example, numerous splicing variants of the mechanosensitive channel PIEZO2 have been found to be expressed in a cell-type-specific manner in sensory ganglia, and these isoforms confer distinct biophysical properties that allow the detection of different types of mechanical stimuli (Szczot et al., 2017). Furthermore, in the case of the mechanosensitive two-pore-domain potassium channel TREK-1, a splicing isoform resulting from an alternative stop codon in the retained intron, TREK-1e, has been identified, and coexpression of TREK-1e with TREK-1 reduces the surface expression of TREK-1 in HEK293T cells (Rinné et al., 2014), this finding indicates that alternative splicing isoforms can regulate the trafficking of the major isoform of a protein. The advent of next-generation sequencing techniques and the development of various computational tools have substantially enhanced our ability to identify alternative splicing at the genome-wide level.

To comprehensively understand the function and regulation of the MT complex, it is crucial to investigate the precise composition of the MT complex, regardless of whether the complex contains other unknown proteins or distinct splicing variants of the known components. Previous studies have identified three splicing variants of PCDH15 (Pepermans et al., 2014) and two splicing variants, featuring alternative translation start sites, of TMC1 (Kawashima et al., 2011). Here, by using publicly available data from mouse auditory hair cells (Li et al., 2018), we identified previously unreported splicing variants of TMC1, LHFPL5, and TMIE, and we then performed RT-PCR and Sanger sequencing to verify the alternative splicing events. The complexity and accuracy of the MT-complex composition revealed by our findings should be carefully considered in future research and in clinical diagnosis of deafness.

## Materials and Methods

### Bioinformatics

Illumina reads of a recently published cDNA-seq library of auditory hair cells of adult mice (Li et al., 2018) were aligned to GRCm38 (mm10) available from Ensembl by using the rapid and sensitive alignment program HISAT2 (Pertea et al., 2016). The Mixture of Isoforms (MISO) Sashimi Plot Feature [Integrative Genomic Viewer (IGV); Broad Institute] was used to visualize alternative splicing events.

### Mice

C57BL/6 background mice were used for experiments. All animal procedures were approved by the University Committee on Research Practices at the Hong Kong University of Science and Technology (the ethics research project number A19005).

### RNA extraction

For use in conventional PCR studies, we dissected the cochlea from four P8 mice of either sex and the cerebrum, cerebellum, cochlea, colon, eye, and testis from three adult (P32–P40) male mice. For quantitative real-time PCR (qRT-PCR), the organ of Corti was dissected from 15 P6 mice of either sex. The distal end at the basal side of the tissues, which was frequently damaged during dissection, was discarded to ensure the integrity of four rows of auditory hair cells, and the remaining part was divided equally into apical, middle, and basal segments (Fig. 3*C*). The same segments from three mice were pooled for one data point. Tissues were homogenized in an RNase-free glass abrader and total RNA was extracted by using a Minibest universal RNA extraction kit (Takara) according to the manufacturer’s protocol. RNA concentration and purity were determined using a Nanodrop 2000 spectrophotometer (Thermo Fisher Scientific).

### PCR and validation of isoforms

To validate the existence of the splicing isoforms of *Tmc1*, *Lhfpl5*, and *Tmie*, primers were designed to target exon 8 and exon 10 of *Tmc1*, exon 1/2 and exon 3 of *Lhfpl5*, or exon 4 and exon 5 of *Tmie* flanking the alternative splicing region; two pairs of unique primers were also designed for verifying the alternative splicing event in exon 14 of *Tmc1* (see details in the legend for Fig. 2*B*). Total RNA was reverse-transcribed into cDNA by using a High-Capacity RNA-to-cDNA kit (Thermo Fisher Scientific). The amount of cDNA used as the PCR template was equivalent to 30 ng of total RNA.

PCR was performed using KAPA HIFI polymerase (Kapa Biosystem). To amplify the alternative splicing isoforms for Sanger sequencing, we used two rounds of PCR, each with 32 cycles of reaction, and an annealing temperature of 60°C. The PCR products from the first round of PCR were separated in 2% agarose gels, and the bands that were of the size predicted for the alternative splicing isoforms were excised and purified for use as the template for the second round of PCR. The final amplified PCR products were purified and subject to Sanger sequencing.

For semiquantitative PCR, the process and conditions were identical to those mentioned above, but only one round of PCR was used and the PCR products were then electrophoretically separated and visualized using a ChemiDoc MP Image System (Bio-Rad).

The primers used in these experiments are listed in Table 1.

**Table 1:** Primers for conventional PCR (including nested PCR and semiquantitative PCR)

Primer name	Sequence
*Tmc1* exon 8 F	AGTGGCCTCGTACTTCCTGTT
*Tmc1* exon 10 R	CGTGCGTTTATTGTCGTAATAGCC
*Tmc1* exon 14 F_1 (primer 1 in Fig. 2B)	TCGTTCATCCTGCAGATG
*Tmc1* exon 15 R (primer 2 in Fig. 2B)	ACGTAAGTGGTCAGGACG
*Tmc1* exon 14 F_1′ (primer 1′ in Fig. 2B)	CACAGGAGCACCCTTTTT
*Tmc1*-Δ9bp-span R (primer 2′ in Fig. 2B)	TGAGACGCACGAATTCCA
*Lhfpl5* exon 1 F_1	CCATCATCTGCTTCAGCCTG
*Lhfpl5* exon 3 R_1	ACCTCGGTTGCTTCAGACTT
*Lhfpl5* exon 1 F_2	GCCTTCAAGACTGCCATGTTC
*Lhfpl5* exon 3 R_2	GAATTGTTGCTGCCAGCACC
*Tmie* exon 4 F	ACCAAGGAGACTGTGGTGTT
*Tmie* exon 5 R	AGCCTCGATCTCCTTCCGC

F, forward primer; R, reverse primer.

### qRT-PCR

To separate the signal of the alternative splicing isoform from that of the constitutive splicing isoform, reverse primers harboring isoform-specific sequences were used. Total RNA was reverse-transcribed into cDNA by using a high-capacity RNA-to-cDNA kit, and the amount of cDNA used as the template for each reaction in qRT-PCR was equivalent to 4.6 ng of total RNA. The primers used are listed in Table 2.

**Table 2:** Primers for qRT-PCR

Primer name	Sequence
*mGAPDH* F	TCACCACCATGGAGAAGGC
*mGAPDH* R	GCTAAGCAGTTGGTGGTGCA
*Tmc1* F	GTCAGTTTGGTTCCTCAGTGGC
*Tmc1* constitutive R	CTAGGTAAGCTGCCGTACGG
*Tmc1* alternative R	GCCAGGCCCTCCGGTAA
*Lhfpl5* F	CCATCATCTGCTTCAGCCTG
*Lhfpl5* constitutive R	CATGAAGGCCCAGCGGAT
*Lhfpl5* alternative R	GACTTCCTCCGTCTGCTCG

F, forward primer; R, reverse primer.

Reaction mixtures were prepared using LightCycler 480 SYBR Green I Master Mix (Roche), as per the manufacturer’s protocol, and qRT-PCR was performed using a LightCycler 480 instrument (Roche). The C_T_ value was determined using a built-in fit-points method. The ratio of concentration of each gene relative to GAPDH gene was calculated using the ΔΔC_T_ method.

### Statistics

All data are shown as mean ± SEM. Statistical analysis was performed using Student’s *t* test, and *p *<* *0.05 was considered statistically significant.

## Results

### *In silico* analysis of alternative splicing in *Tmc1*, *Lhfpl5*, and *Tmie*

Owing to the development of second-generation sequencing techniques, RNA-seq data of rare cells such as auditory hair cells have now become available. By using a recently published cDNA-seq library of auditory hair cells of adult mice (Li et al., 2018), we analyzed alternative splicing in the genes *Tmc1*, *Lhfpl5*, and *Tmie* (see Materials and Methods) and obtained the following results.

**Figure 1.**
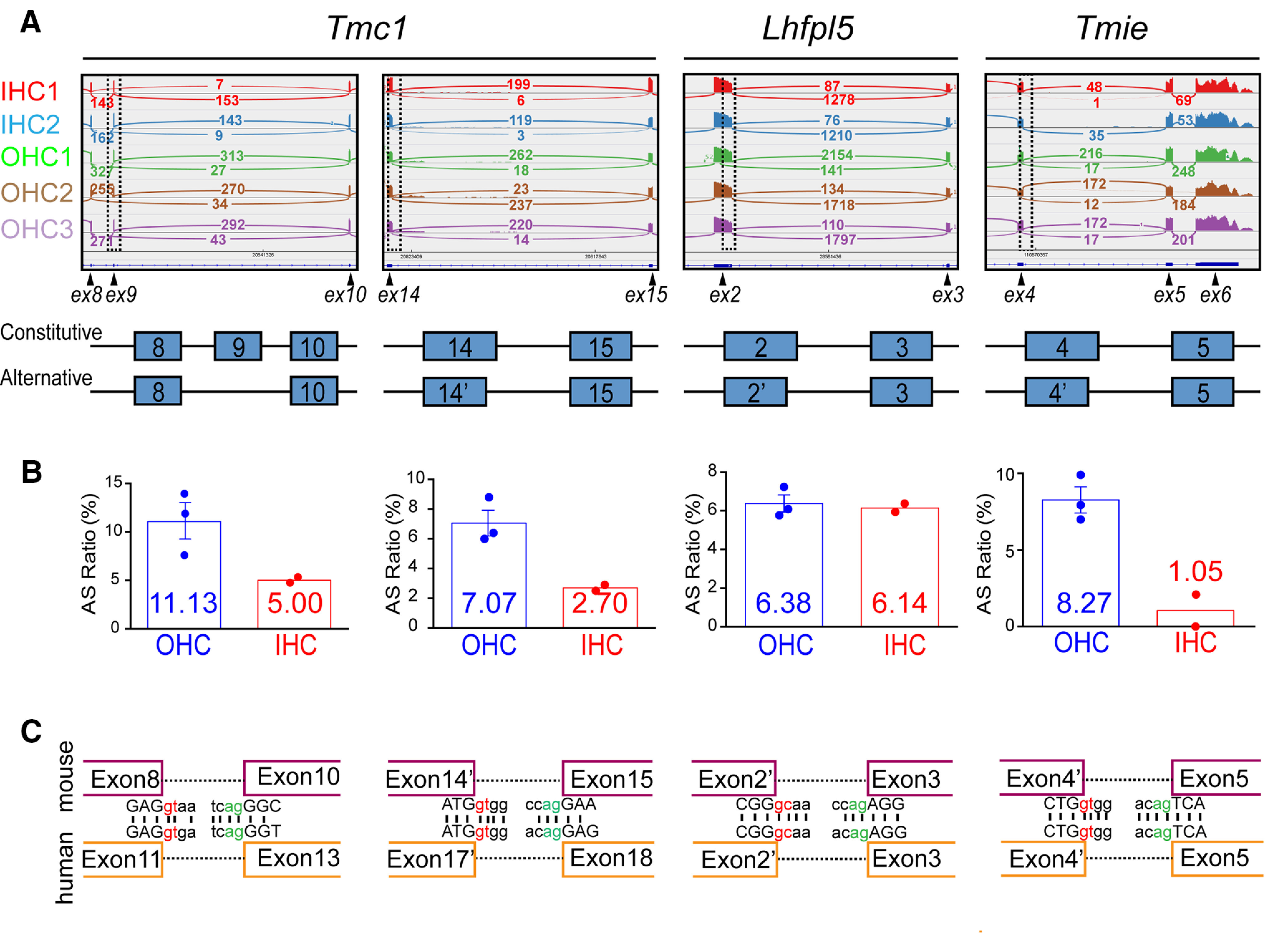
*In silico* analysis of alternative splicing in *Tmc1*, *Lhfpl5*, and *Tmie*. ***A***, upper panel, Sashimi plots showing coverage of exons and junctions. Individual biological replicates are indicated using different colors; rectangular boxes: regions where alternative splicing events occur. Exons (ex) corresponding to peaks of reads are indicated below. Lower panel, Schematic of constitutive and alternative splicing isoforms; lines: introns. ***B***, Corresponding ratios of alternative splicing (AS) events to total splicing (constitutive and alternative) events illustrated in panel ***A***. AS ratios in IHCs and OHCs are shown; AS ratio = number of junctions using alternative splicing sites/number of junctions using both constitutive and alternative splicing sites. ***C***, Conservation in mice and humans of the corresponding splicing sites for the four alternative splicing events depicted in panel ***A***. Exons upstream and downstream of splicing sites are represented in purple (mouse) or orange (human). Donor sites (*gc* for splicing event in *Lhfpl5* and *gt* for others) are shown in red, acceptor sites (*ag*) in green. Adjunct sequences in exons are uppercased, intronic sequences are lowercased. Vertical bars, identical nucleotides.

First, we found that *Tmc1* starts at exon 1 (and then skips exon 2) or alternatively starts at exon 2 (data not shown) as previously reported (Kawashima et al., 2011), the two isoforms are designated as TMC1A (starting at exon 1) and TMC1B (starting at exon 2). TMC1A is considerably more abundant than TMC1B (ratio ∼95:5) and is therefore considered to be the canonical form (Yamaguchi et al., 2020). More importantly, we identified two previously undescribed alternative splicing events: exon 9 skipping and alternative 3′ splicing in exon 14 at chr19:20 823 987 (Figs. 1*A*, 4).

**Figure 2.**
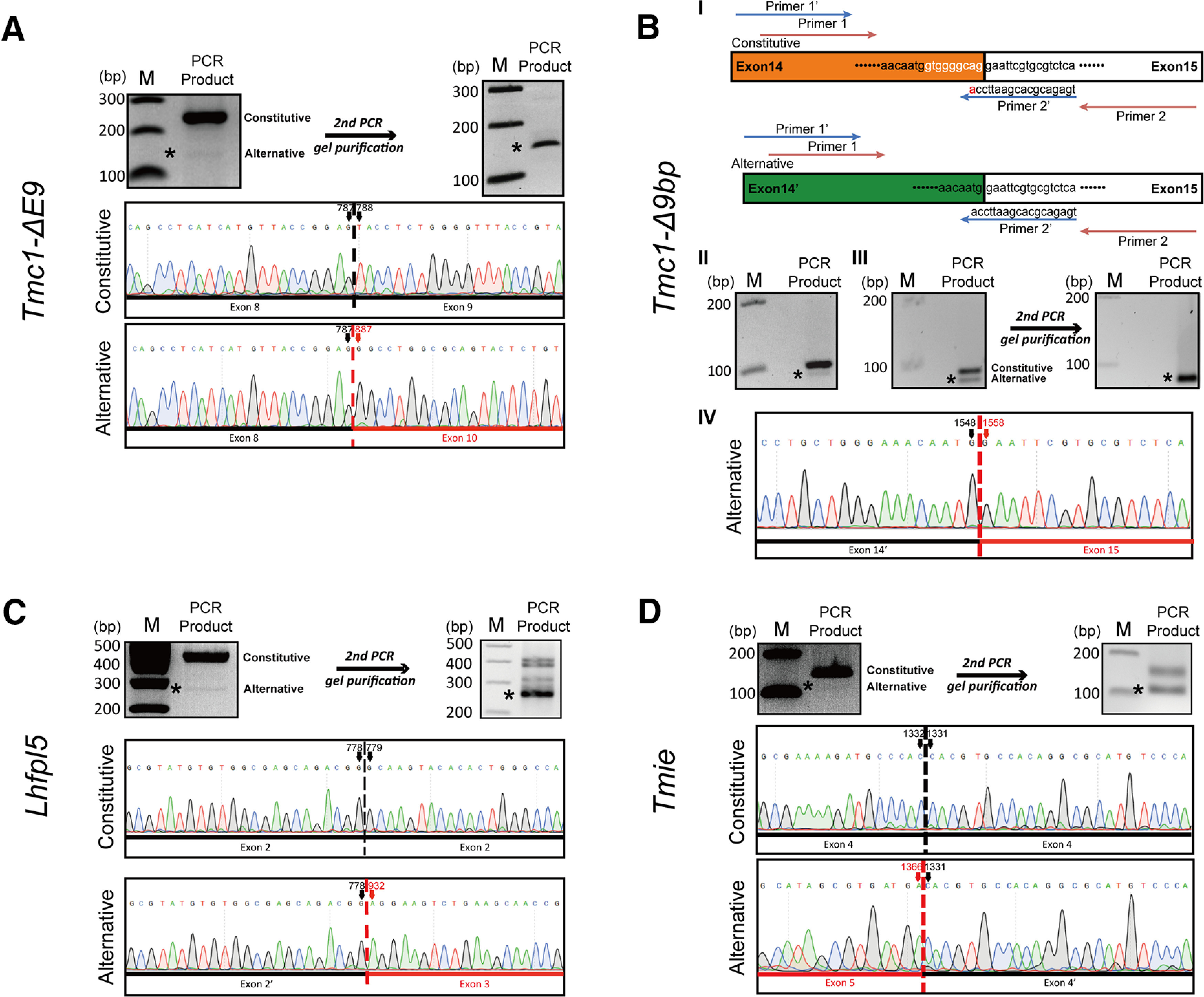
Validation of splicing events through Sanger sequencing. ***A***, Mouse cochlear cDNA was prepared through reverse transcription and used in the first round of PCR with primers flanking predicted splicing sites in *Tmc1* (exon 9 skipping). After PCR products were separated using agarose-gel electrophoresis (left, upper panel), the band at the predicted size for the constitutive splicing variant was excised, purified, and subject to Sanger sequencing. The band at the predicted size for alternative splicing variants was excised, purified, and used as the template for the second round of PCR (right, upper panel); the final PCR products in the second round were purified and subject to Sanger sequencing. *, alternative splicing isoforms. Electropherograms are presented in middle and lower panels; numbers at the top of electropherograms indicate nucleotide positions in the mRNA sequence annotated by NCBI. ***B***, Validation of *Tmc1* splicing: 9-bp skipping at 3′ end of exon 14. Mouse cochlear cDNA was prepared through reverse transcription and used as the template for the PCR in panel ***II*** and for the first round of PCR in panel ***III***; the band at the predicted size for the alternative splicing variant in the first round of PCR in panel ***III*** was excised, purified, and used as the template for the second round of PCR. Primers 1/2 (orange) and 1′/2′ (blue) were used for PCR in panels ***II***, ***III***, respectively; primer 2′ contained one base mismatched to the constitutive splicing isoform (panel ***I***). The PCR product of the second round of PCR in panel ***III*** was inadequately long for clear sequencing of the region around the alternative splicing site, and therefore the product was slightly extended by cloning it into pcDNA3 plasmid before Sanger sequencing. ***C***, ***D***, Experiments similar to ***A*** performed for *Lhfpl5* (***C***) and *Tmie* (***D***). P8 mice were used in all experiments; M, molecular-weight size markers.

**Figure 3.**
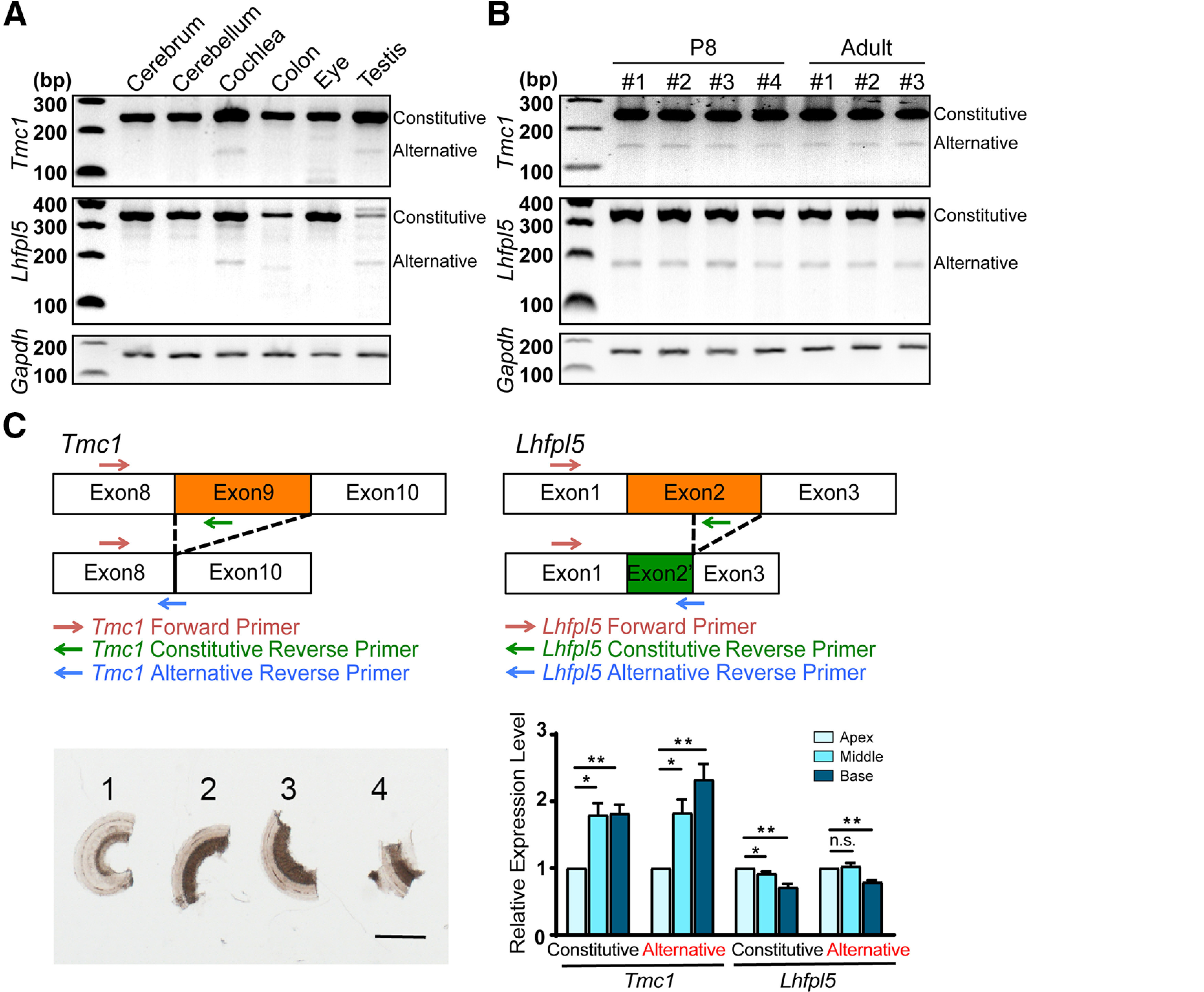
Differential expression of TMC1 and LHFPL5 isoforms. Total RNA was extracted from different tissues from P31–P33 mice (***A***) or cochleae from P8 and adult (P32–P40) mice (***B***), and mRNA was reverse-transcribed into cDNA by using oligo-dT primers. Approximately 30 ng of starting cDNA from each tissue was used as the PCR template, and final amplified products were separated on agarose gels. Bands representing constitutive and alternative splicing isoforms are labeled. GAPDH, loading control. ***C***, upper panel, Schematic of primers used in qRT-PCR of constitutive and alternative splicing isoforms. Lower panel, left, Organ of Corti from P6 mice was dissected into four segments. Segments 1–3: apical, middle, and basal parts of organ of Corti. Segment 4 was discarded because it was frequently damaged during dissection. Samples were fixed to flatten the coiled segments and thereby improve their imaging. Scale bar: 100 μm. Lower panel, right, Results of qRT-PCR. *N* = 5 independent biological replicates. From left to right: for *Tmc1*, *p =* *0.010, **p =* *0.003, *p =* *0.015, **p =* *0.005; for *Lhfpl5*, *p =* *0.049, **p =* *0.005, n.s., not significant, **p =* *0.002.

**Figure 4.**
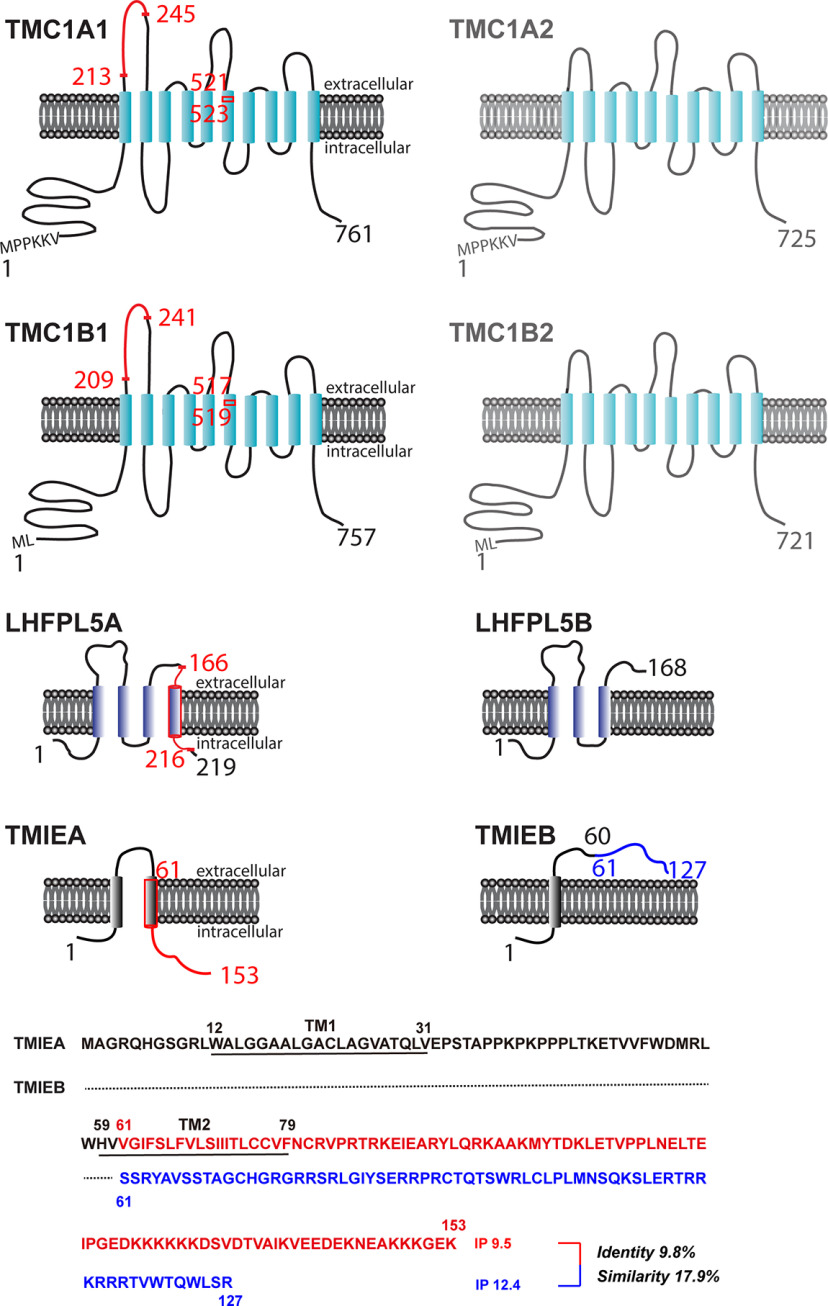
Schematic depiction of splicing variants of TMC1, LHFPL5, and TMIE. Transmembrane domains are shown as columns, and intracellular/extracellular domains are indicated by lines. For each protein, alternatively spliced-out regions and their starting or ending amino acids are shown in red. For TMIEB, the C terminus generated by alternative splicing and reading-frame shift is highlighted in blue. Bottom panel, C-terminal sequences of TMIEB (blue) and TMIEA (red); dotted line, amino acids identical to that in TMIEA; IP, isoelectric point.

Second, we identified an alternative 3′ splicing site in exon 2 of *Lhfpl5* at chr17:28580014 (Fig. 1A). Intriguingly, this alternative splicing site was fortuitously detected and suggested as a cryptic splicing site in *Lhfpl5^hscy-2J^* mice, in which the normal splicing donor-recognition site of exon 2 was disrupted as a result of N-ethyl-N-nitrosourea-induced mutagenesis and hearing was severely damaged (Longo-Guess et al., 2007). We designated the full-length, constitutive splicing LHFPL5 isoform as LHFPL5A and the alternative splicing isoform as LHFPL5B (Fig. 4). LHFPL5A is substantially more abundant than LHFPL5B (Fig. 1*A*, ratio ∼93:7) and is thus regarded as the canonical form.

Third, we also identified an alternative 3′ splicing site in *Tmie* exon 4 at chr9:110870665 (Fig. 1*A*). The constitutive and alternative splicing isoforms of TMIE (ratio ∼95:5) were designated as TMIEA and TMIEB, respectively (Fig. 4).

All the newly identified splicing events showed the highest junction coverage and could be reproduced in at least four out of the five batches of biological replicates in the study of Li et al. (2018; Fig. 1*A*). Interestingly, both of the newly identified alternative splicing events in TMC1 and the alternative splicing event in TMIE appeared to feature a higher ratio in OHCs than in inner hair cells (IHCs; Fig. 1*B*). The alternative splicing ratio was determined by dividing the number of alternative splicing junctions by the total number of junctions (alternative and constitutive). By contrast, the alternative splicing ratio of LHFPL5 was similar in OHCs and IHCs (Fig. 1*B*). Because RNA-seq data for human inner ear are unavailable, we analyzed the conservation of the splicing donor/receptor sites and the nearby adjunct nucleotides between mice and humans. The high conservation observed indicates that the alternative splicing events might occur in humans as well (Fig. 1*C*).

One concern regarding RNA-seq is that the reverse-transcription process in this technique might disproportionally amplify shorter isoforms because the dT primer targets the poly-A region and longer isoforms are more difficult to reverse-transcribe than shorter isoforms; this could lead to overestimation of the ratio of alternative/constitutive splicing isoforms. A similar concern could apply to PCR amplification of cDNA. However, the length differences between the transcripts of the alternatively and constitutively spliced isoforms in this study were small (length ratios: TMC1B2/TMC1B1 = 0.973; LHFPL5B/LHFPL5A = 0.88; TMIEB/TMIEA = 0.988) and presumably would have minimally affected the analysis of the ratio of alternative/constitutive splicing isoforms.

### Validation of splicing events in *Tmc1, Lhfpl5*, and *Tmie* through Sanger sequencing

To eliminate potential artifacts in *in silico* analyses, alternative splicing identified by analyzing RNA-seq data must be verified using Sanger sequencing. Here, sequencing results obtained using primers flanking the predicted alternative splicing sites in *Tmc1* (exon 9 skipping; Fig. 2*A*), *Lhfpl5* (Fig. 2*C*), and *Tmie* (Fig. 2*D*) confirmed the existence of three splicing events in *Tmc1* (exon 9 skipping), *Lhfpl5*, and *Tmie*. However, this approach involving the use of primers flanking the predicted alternative splicing site cannot be used to verify the alternative splicing event in exon 14 of *Tmc1*; this is because the alternative splicing removes only nine nucleotides (Figs. 1*A*, 2*B*), and the alternative splicing isoform therefore cannot be resolved without contamination by the predominant constitutive splicing isoform in the agarose-gel electrophoresis performed before Sanger sequencing, although the PCR products were as short as ∼100 bp and showed clear separation (Fig. 2*BI*,*II*). Thus, as an alternative, we used the strategy of primer-template mismatch (Kwok et al., 1990; Stadhouders et al., 2010).

In principle, every mismatch between a primer and template reduces priming efficiency and overall PCR product yield; however, mismatches in the last five bases of the 3′-end region of a primer produce a considerably stronger effect than do mismatches located more toward the 5′ end (Kwok et al., 1990; Stadhouders et al., 2010). Intriguingly, this undesirable effect of primer-template mismatch can be useful in basic research and molecular diagnostics: it enables differential detection of nucleic acid sequences featuring small differences, such as in the detection of single nucleotide polymorphisms and in allele-specific PCR (Stadhouders et al., 2010). Here, we designed a special reverse primer for *Tmc1* (Figure 2*BI*, primer 2′) that crosses over the alternative splicing site by two nucleotides and therefore features a one-nucleotide mismatch with the constitutive splicing isoform while perfectly matching the alternative splicing isoform; notably, the one-nucleotide mismatch drastically reduced the PCR product yield of the constitutive splicing isoform (Fig. 2*BIII* vs *BII*) and the contamination of the alternative splicing isoform by the constitutive splicing isoform. This approach allowed us to isolate and sequence the alternative splicing isoform (Fig. 2*BIII*,*IV*). Importantly, when the pure constitutive splicing isoform was used as the template, primers 1′/2′ did not generate the PCR product of the alternative splicing isoform (data not shown), which suggests that primer 2′ cannot loop out the 5′-terminal nine bases in exon 14 of *Tmc1* to artificially generate the PCR product of the alternative splicing isoform.

### Differential expression of TMC1 and LHFPL5 isoforms

Splicing isoforms of proteins are widely suggested to display tissue-specific or cell-specific expression patterns. To test whether TMC1 and LHFPL5 splicing isoforms exhibit tissue-specific expression, we performed semiquantitative RT-PCR to examine the expression of the isoforms in various tissues (Fig. 3). In agreement with previous work suggesting the presence of TMC1 mRNA in six tested tissues (Keresztes et al., 2003), the constitutive splicing TMC1 isoform was amplified from the cDNAs generated from these tissues (Fig. 3*A*). Furthermore, these tissues were also found to contain LHFPL5 mRNA (Fig. 3*A*). Interestingly, a lower band representing the alternatively spliced isoform of TMC1 and LHFPL5 was detected only in the cochlea and testis. These results suggest that TMC1 and LHFPL5 isoforms potentially perform tissue-specific functions.

We next examined the developmental profile of TMC1 and LHFPL5 splicing isoforms in the cochlea. The cDNAs generated from four mouse pups (at P8) and three adult mice (two at P32 and one at P40) were amplified using primers for TMC1 and LHFPL5 isoforms. Our results showed that the TMC1 and LHFPL5 alternatively spliced isoforms, similar to the constitutively spliced isoforms, appeared at P8 and remained detectable at adulthood (Fig. 3*B*), this suggests that the TMC1 and LHFPL5 spliced isoforms could play critical roles throughout life in hair cells.

We also assessed the tonotopic gradient of the constitutive and alternative splicing isoforms of TMC1 and LHFPL5. Considering that the tonotopic gradient might be too small to allow detection by using semiquantitative methods, we performed the comparatively more sensitive qRT-PCR assay with primers specifically targeting the constitutive and alternative splicing isoforms. Our results suggest the existence of an upward apicobasal gradient for the constitutive splicing TMC1 isoform in the organ of Corti (Fig. 3*C*), this is consistent with the previously reported tonotopic gradient of TMC1 protein in hair bundles (Beurg et al., 2018). By contrast, the constitutive splicing LHFPL5 isoform displayed a modest downward apicobasal gradient (Fig. 3*C*). Moreover, for both TMC1 and LHFPL5, the tonotopic gradient of the alternative splicing isoforms was the same as that of the constitutive splicing isoforms (Fig. 3*C*), which suggests that an unbiased splicing machinery operates throughout the organ of Corti.

### Schematic protein topology of mouse TMC1, LHFPL5, and TMIE isoforms

To help visualize the potential functional consequences of the alternative splicing events reported here, we schematically depicted the topologies of the identified TMC1, LHFPL5, and TMIE isoforms (Fig. 4). The topology of TMC1 is based on two recent homology models (Ballesteros et al., 2018; Pan et al., 2018) rather than on an early model based on hydropathy and epitope accessibility (Labay et al., 2010), the topologies of LHFPL5 and TMIE are based on a 3D structure (Ge et al., 2018) and a predicted model (Zhao et al., 2014), respectively.

TMC1A (NCBI accession: XP_036017315) and TMC1B (NCBI Accession: NP_083229), the TMC1 isoforms starting at exon 1 (and skipping exon 2) and exon 2, respectively, differ only in five residues at the extreme N terminus: MPPKKV in TMC1A and ML in TMC1B (Fig. 4). We designated the full-length, constitutive splicing isoforms of TMC1A and TMC1B as TMC1A1 and TMC1B1 and the alternative splicing isoforms as TMC1A2 and TMC1B2. Both TMC1A1 and TMC1B1 might undergo both of the newly identified alternative splicing events: (1) exon 9 skipping, which deletes amino acids 213–245 (TMC1A) or amino acids 209–241 (TMC1B) in the first extracellular loop; and (2) alternative splicing at exon 14, which deletes amino acids 521–523 (TMC1A) or amino acids 517–519 (TMC1B) in the sixth transmembrane domain. Although these splicing events might occur separately in one transcript, for simplicity, we have tentatively designated TMC1A2 and TMC1B2 as including both the alternative splicing events. The spliced-out segments in TMC1A or TMC1B are highlighted by solid red lines in Figure 4.

LHFPL5 features two isoforms: (1) LHFPL5A (NCBI Accession: NP_080847), full-length, constitutive splicing isoform; and (2) LHFPL5B, in which alternative splicing occurs at exon 2 and results in the deletion of amino acids 166–216, a region containing part of the second extracellular loop, the fourth transmembrane domain, and almost the entire C terminus (Fig. 4).

TMIE is also expressed as two isoforms: (1) TMIEA (NCBI Accession: NP_666372), full-length, constitutive splicing isoform; and (2) TMIEB, in which alternative splicing occurs at exon four and leads to a reading-frame shift starting at amino acid 61 in the extracellular domain, thus producing a final protein product containing one transmembrane domain and a total of 127 amino acids (Fig. 4).

## Discussion

Among the four known proteins assembled in the MT complex, namely PCDH15, TMC1, LHFPL5, and TMIE, PCDH15 has been reported to feature three isoforms: CD1, CD2, and CD3 (Ahmed et al., 2001; Webb et al., 2011; Pepermans et al., 2014). However, little is known regarding the alternative splicing of the other 3 MT-complex components. In the case of TMC1, previous studies have suggested the existence of two isoforms with alternative start codons in mice (TMC1A and TMC1B; Fig. 4; Kawashima et al., 2011). Here, we report two previously unidentified alternative splicing events in *Tmc1*, exon 9 skipping and alternative 3′ splicing in exon 14 (Fig. 4), and we also identify alternative splicing events in *Lhfpl5* and *Tmie* (Fig. 4). Notably, the alternative splicing sites in the three genes are highly conserved in mice and humans, which suggests that the alternative splicing events are highly likely to occur in humans as well.

Intriguingly, both *Tmc1* and *Lhfpl5* display a tissue-specific splicing pattern in the cochlea and testis. Because TMC1 and LHFPL5 play critical roles in hair-cell function, alternative splicing might be functionally important in the case of both proteins (see below). By contrast, the roles of TMC1 and LHFPL5 in the testis have not been closely examined, although knocking TMC1 or LHFPL5 does not appear to affect fertility (Longo-Guess et al., 2007; Kurima et al., 2015). The functions in the testis of these two proteins (of both their constitutive and alternative splicing isoforms) warrant further investigation.

Our data indicate an upward apicobasal gradient for the constitutively spliced TMC1 mRNA in the organ of Corti (Fig. 3*C*), this roughly agrees with the tonotopic gradient of TMC1 protein expression in hair bundles (Beurg et al., 2018). By contrast, the constitutively spliced LHFPL5 mRNA displayed a modest downward apicobasal gradient (Fig. 3*C*); however, further investigation is required to determine whether and how this gradient corresponds to global and hair-bundle LHFPL5 protein expression in hair cells. Moreover, the tonotopic gradient of the alternative splicing isoforms of both genes was the same as that of the constitutive splicing isoforms (Fig. 3*C*), and the lack of a tonotopic gradient in the splicing ratio of the two genes suggests the existence of an unbiased splicing machinery across the entire organ of Corti.

What are the potential functions of the alternative splicing isoforms of the MT-complex proteins? The three splice isoforms of PCDH15, CD1, CD2, and CD3, differ only in their cytoplasmic domains, as a result of exclusion and inclusion of exons through alternative splicing (Webb et al., 2011; Pepermans et al., 2014), and whereas the isoforms are functionally redundant at an early developmental stage in mice, CD2 becomes critical in mature hair cells (Pepermans et al., 2014). Conversely, the difference in the cytoplasmic domain of the isoforms regulates the interaction between PCDH15 and TMIE (Zhao et al., 2014).

The two isoforms of TMC1 featuring alternative translation initiation sites (TMC1A and TMC1B) differ only within the first 5 amino acid residues at the N terminus (Fig. 4), and although the functional difference between the two isoforms remains unclear, it is expected to be minor (Yamaguchi et al., 2020). Both isoforms can rescue the transducer current in TMC1-knock-out mice (Kawashima et al., 2011; Nist-Lund et al., 2019; Cunningham et al., 2020). Exon 9 skipping deletes amino acids 213–245 in TMC1A (amino acids 209–241 in TMC1B) in the first putative extracellular loop, and this 33-aa segment contains an alternative N-linked glycosylation site NXG, N_237_FG, in TMC1A (Lowenthal et al., 2016). Because N-glycosylation is involved in protein folding and function, splicing out this segment might affect TMC1 folding and function. Furthermore, alternative splicing at exon 14 deletes amino acids 521–523 in TMC1A (amino acids 517–519 in TMC1B) in the sixth transmembrane domain (Fig. 4), this could affect hearing by disrupting TMC1 channel function: the spliced-out three residues, V521GQ, appear to form part of the channel pore in the published homology models (Ballesteros et al., 2018; Pan et al., 2018).

Interestingly, the GluR6a subunit of ionotropic glutamate receptors has been shown to promote cell-surface expression of its splicing variant GluR6b and several GluR5 splicing variants (Jaskotski et al., 2004). Thus, it will be of interest to investigate how the alternative splicing isoforms of TMC1 modulate the trafficking of the constitutive splicing isoform of TMC1.

Alternative splicing in exon 2 of LHFPL5 generates the LHFPL5B isoform; the splicing deletes amino acids 166–216, a region that includes part of the second extracellular loop, the fourth transmembrane domain, and almost the entire C terminus (Fig. 4). Notably, LHFPL5B by itself failed to support any hearing function: profound deafness was recorded in homozygous *Lhfpl5^hscy-2J^* mice (*Lhfpl5^B/B^* mice expressing the LHFPL5B isoform; Longo-Guess et al., 2007). Moreover, the LHFPL5B gene (resulting from the hscy-2J mutation) was suggested to be recessive because heterozygous *Lhfpl5^hscy-2J^* mice showed normal hearing. However, this notion remains to be confirmed because it did not account for the finding that the wild-type allele in the heterozygous mice produces not only LHFPL5A through canonical splicing, but also LHFPL5B through alternative splicing: in principle, the wild-type allele could produce a sufficient amount of LHFPL5B and thereby mask the dominant (positive or negative) effect of LHFPL5B generated by the mutant allele (hscy-2J). Therefore, how LHFPL5B affects LHFPL5A protein function remains unelucidated.

Alternative splicing in exon four of TMIE, which generates TMIEB, causes a reading-frame shift starting after amino acid 60 in the extracellular domain and produces a final protein product that contains one transmembrane domain and is 127 aa long (Fig. 4). The C terminus (amino acids 61–127) in TMIEB shares little homology with the TMIEA C terminus (amino acids 61–153; 9.8% identify and 17.9% similarity) and does not appear to contain any transmembrane helices according to TMpred (data not shown), this algorithm is used for statistical analysis of TMbase, which stores naturally occurring transmembrane proteins (Hofmann and Stoffel, 1993). Because the C-terminal half of TMIEA has been found to be critical for binding to TMC1, PIP2, PCDH15, and LHFPL5 (Zhao et al., 2014; Cunningham et al., 2020), TMIEB might potentially not support any normal hearing function. TMIEB could act as a dominant-negative regulator of TMIEA function if TMIEB, particularly its N-terminal 60 aa shared with TMIEA, still binds to a known or unknown component of the MT complex. Whether the N terminus of TMIE binds to any MT-complex component is currently unknown, although the first 28 aa of TMIE appear to mediate the MT-channel response to mechanical cues (Cunningham et al., 2020). Interestingly, the C terminus of TMIEB, similar to that of TMIEA, is also highly positively charged (Fig. 4) and might interact with PIP2 (Cunningham et al., 2020).

In summary, we have identified previously unreported splicing variants of TMC1, LHFPL5, and TMIE, the pivotal molecules forming the hair-cell MT machinery. Our findings reveal the potential complexity of the MT-complex composition and provide valuable guidance for future research on the function, regulation, and trafficking of TMC1, LHFPL5, and TMIE. Furthermore, our study could help direct the clinical diagnosis of hearing loss related to aberrant splicing of *TMC1*, *LHFPL5*, and *TMIE*. The key question to address next is the physiological function of the alternative splicing isoforms of these proteins critical for normal hearing.

## Synthesis

Reviewing Editor: Leonard Maler, University of Ottawa Decisions are customarily a result of the Reviewing Editor and the peer reviewers coming together and discussing their recommendations until a consensus is reached. When revisions are invited, a fact-based synthesis statement explaining their decision and outlining what is needed to prepare a revision will be listed below. The following reviewer(s) agreed to reveal their identity: NONE.

Editorial comments

Both reviewers find this to be an interesting manuscript with some useful new information. However they also describe a number of serious difficulties that must be addressed before this Ms is acceptable for publication in eNeuro.

Notably, Reviewer #1 asks whether the TMC1 variants are used and functional.

This problem might be addressed by using methods to express transcripts in hair cells or other cells. Further, the quality of the image of the dissected cochlea in Fig. 3C is poor and there is no reason to omit the large basal piece.

I have included the full and detailed reviews and the authors should carefully address all the points raised. Please provide a red lined Ms with the changes highlighted as well as a detailed rebuttal.

Reviewer #1

This manuscript describes alternatively spliced variants of three proteins, TMC1, TMIE and LHFPL5, thought to be an integral part of the mechanotransducer channel in cochlear hair cells. Three methods were applied: bioinformatics analysis of published cDNA library (Li et al 2018) from adult inner and outer hair cells, conventional PCR on cochlear hair cells and qRT-PCR. The variants in LHFPL5 and TMIE severely shorten or alter the protein sequence and it seems likely that they are non-functional. For the TMC1, the presence of two start sites due to skipping of exon 2, as described by Yamaguchi et al (2019), was confirmed. The two new TMC1 splice sites are the most interesting discovery, one deleting exon 9, residues 209-241 in the first extracellular loop and the other deleting residues 517-519 in TM6 at the entrance to proposed channel pore. These are expressed more in outer hair cells at 7 - 11 percent of total TMC1 (Fig. 1B), and both unspliced and spliced transcripts increase from cochlear apex to base (Fig. 3C).

New information on TMC1 is important and topical but significant criticisms relate to whether these TMC1 variants are used or are functional. Due to its putative location, the 517-519 variant may deleteriously affect ion permeation. Some attempt must be used to address this problem by using methods to express transcripts in hair cells or other cells. There were also concern about the quality of the image of the dissected cochlea in Fig. 3C: it looks poor and there is no reason to omit the large basal piece. It would have been useful to have some statement about whether the relative expression of the new TMC1 splice variants changed with age as the start sites (A or B) are thought to do (Yamaguchi et al 2019). Could the q-RT-PCR be used to validate the relative difference between spliced and unspliced TMC1 as inferred from analysis previously published data?

Reviewer #2

Regarding manuscript eN-NWR-0381-20, there are many issues that need to be addressed by the author(s). The authors report alternative splice isoforms of mouse Tmc1, Lhfpl5 and Tmie, encoding three of the proteins that are part of the mechanotransduction complex at the tips of stereocilia protruding from the apical surface of inner ear hair cells. These three genes were first discovered when variants of the human orthologous genes were reported to be associated with deafness. This historical context is important to understand the importance of these genes for normal hearing. The original references for these three genes should be mentioned and cited in the manuscript.

Line 45, The authors state that "For example, TMC1 mRNA expression is tissue specific..." Is the TMC1 gene only expressed in the inner ear or is Tmc1 expressed in many different tissues?

Line 59, What is meant by "... a prematurely stopped splicing isoform, ..."? This seems like clumsy language.

Figure 1, panel A, The Sashimi plots for exons of TMC1 and Tmie are drawn from right to left for increasing exon number while Lhfp15 is from left to right for increasing exon numbers. Below the Sashimi plots are diagrams of the three genes, all drawn with increasing exon numbers from left to right. Sashimi plots should correspond to the drawing of the exons with increasing exon numbers from left to right.

Figure 1, panel B, is said to show "Alternative splicing ratios of IHCs and OHCs." Splicing ratios of what? What exactly is the numerator and denominator of the ratio and how were these numbers obtained? The information in the Materials and Methods (line 84) and elsewhere in the text is not adequate. The figure legend and figures themselves should be sufficiently explanatory to understand the data. Make clear in the legend how this ration was calculated. In the text, indicate the types of errors introduced by reverse transcription of mRNA and PCR amplifying the cDNA and how these problems could impact the ratio calculations?

Line 88, provide the animal protocol number and the investigator’s name in the published manuscript.

Today, the state of the art for studying alternative spliced transcripts are full-length reads on a PacBio instrument or Oxford Nanopore. Consider mentioning the pitfalls and shortcomings of identifying alternative splice isoforms from short cDNA reads.

Line 240, Discussion, describe exactly what are CD1, CD2 and CD3 isoforms of PCDH15 and give the reference where these isoforms were reported at this point in the Discussion.

Figure 1, panel C, Define the terms used conventional donor and acceptor site. Are splice sites always gt and ag? And what is an "adjunct sequence"? A vertical line is not a "dash".

Figure 2, panel A, label the lanes for the two images. What is in each lane?

How do you know the intense band is "unspliced mRNA" and not an artifact of contaminating genomic DNA contamination? What were the templates for each lane? Panel C, the data is not referred to as a "peak map"? Consider electropherogram.

Figure 3, panel A, Same issue as above. Why are authors convinced the dense bands are unspliced mRNA and not largely if not entirely genomic DNA contamination? This would be most unusual if an unspliced mRNA is a predominant transcript recovered. How would you explain such high levels of unspliced mRNA? Also, some of the "spliced" transcript bands shown in the gel image are much too weak to be believable.

• Figure 4, There are a few different models for the number and structure of the TMC1 gene, two of which were published in the last few years, one from Harvard and two others from groups at the NIH. These models are cited on lines 215-216. One that was left out was published 10 years ago by V. Labay (PMCID: PMC3005947). If they differ from one another, what model was used to draw the topology of TMC1, for example, and for the other proteins as well. Provide accession numbers for the residues for all of the proteins in Figure 4 so that the residue numbers can be documented.
